# Inhibition of the Peroxygenase Lytic Polysaccharide Monooxygenase by Carboxylic Acids and Amino Acids

**DOI:** 10.3390/antiox11061096

**Published:** 2022-05-31

**Authors:** Erik Breslmayr, Peter Poliak, Alen Požgajčić, Roman Schindler, Daniel Kracher, Chris Oostenbrink, Roland Ludwig

**Affiliations:** 1 Institute of Food Technology, Department of Food Science and Technology, University of Natural Resources and Life Sciences (BOKU), 1190 Vienna, Austria; erik.breslmayr@gmail.com (E.B.); alen.pozgajcic@gmail.com (A.P.); roman.schindler@students.boku.ac.at (R.S.); roland.ludwig@boku.ac.at (R.L.); 2 Institute of Molecular Modeling and Simulation, University of Natural Resources and Life Sciences (BOKU), 1190 Vienna, Austria; peter.poliak@boku.ac.at (P.P.); chris.oostenbrink@boku.ac.at (C.O.); 3 Department of Chemical Physics, Faculty of Chemical and Food Technology, Slovak University of Technology, 812 37 Bratislava, Slovakia; 4 Department of Biochemical Engineering, Faculty of Food Technology and Biotechnology, University of Zagreb, 10000 Zagreb, Croatia; 5 Institute of Molecular Biotechnology, Graz University of Technology, Petersgasse 14, 8010 Graz, Austria

**Keywords:** density functional theory, effector, inhibitor, lytic polysaccharide, monooxygenase, molecular dynamics simulations, peroxygenase, activity, photometry, turbidimetry, quantum mechanical calculations

## Abstract

Lytic polysaccharide monooxygenases (LPMOs) are widely distributed in fungi, and catalyze the oxidative degradation of polysaccharides such as cellulose. Despite their name, LPMOs possess a dominant peroxygenase activity that is reflected in high turnover numbers but also causes deactivation. We report on the influence of small molecules and ions on the activity and stability of LPMO during catalysis. Turbidimetric and photometric assays were used to identify LPMO inhibitors and measure their inhibitory effect. Selected inhibitors were employed to study LPMO activity and stability during cellulose depolymerization by HPLC and turbidimetry. It was found that the fungal metabolic products oxalic acid and citric acid strongly reduce LPMO activity, but also protect the enzyme from deactivation. QM calculations showed that the copper atom in the catalytic site could be ligated by bi- or tridentate chelating compounds, which replace two water molecules. MD simulations and QM calculations show that the most likely inhibition pattern is the competition between the inhibitor and reducing agent in the oxidized Cu(II) state. A correlation between the complexation energy and the IC_50_ values demonstrates that small, bidentate molecules interact strongest with the catalytic site copper and could be used by the fungus as physiological effectors to regulate LPMO activity.

## 1. Introduction

Lytic polysaccharide monooxygenases (LPMOs) catalyze the cleavage of various biopolymers, including cellulose [1,2] and chitin [3]. LPMOs employ a yet-unidentified radical oxygen species to catalyze the regioselective oxidation of the C4 or C1 carbon atoms in glycosidic bonds, resulting in strand breaks that promote the activity of associated cellulolytic enzymes [4]. Recent studies have shown that bacterial and fungal LPMOs preferably use hydrogen peroxide (H_2_O_2_) rather than oxygen as a cosubstrate, indicating a dominant peroxygenase reactivity [5,6,7]. While such reactivity is of potential interest for biotechnological applications, it can also result in detrimental autooxidation reactions that lead to enzyme deactivation, especially in the absence of saturating substrate concentrations [7,8,9]. Enhancing LPMO stability by optimizing process conditions or enzyme-engineering approaches is critical to improving the utility of these enzymes for industrial applications.

It is noteworthy that a relationship between the activity and stability of LPMOs and the type, concentration and pH-dependence of the reductants required to initiate LPMO activity was observed [10,11,12,13]. Frommhagen et al. showed that increasing the pH from 3 to 6 yields higher product formation [10]. Furthermore, it was observed that at a pH of 3–4, the product release leveled off much earlier than at pH 5–6. For pH values above 6.0, the authors mentioned that high catalytic turnover was observed, albeit with concomitantly fast inactivation of LPMO. In addition, the reductants used were not stable at higher pH values [10]. Hegnar et al. showed that the reducing agent 2,3-dihydroxybenzoic acid, which has a higher redox potential than the frequently used ascorbic acid, could not fuel the LPMO reaction with oxygen as cosubstrate at pH 6–7 [11]. However, when employing H_2_O_2_ as a cosubstrate, catalysis was observed. Reactions without H_2_O_2_ started to work at pH 8–9, which was attributed to a reduced redox potential and production of H_2_O_2_ by the reductant. The authors concluded that O_2_ cannot bind to the reduced copper and that H_2_O_2_ is the natural cosubstrate. In reactions with ascorbic acid as a reductant, LPMOs are catalytically active because the instability of ascorbic acid promotes the formation of H_2_O_2_, which fuels LPMO catalysis [11].

In addition, not only the pH but also the buffer species affect the activity of LPMO. The LPMO-catalyzed oxidation of 2,6-dimethoxyphenol (2,6-DMP) or hydrocoerulignone to coerulignone was very slow in oxalate or histidine buffers. Furthermore, the difficulties in detecting LPMO activity by these photometric assays indicated that the presence of substances such as histidine inhibits LPMO activity in culture media [14]. However, no systematic study on the effect of small molecules and ions on LPMO activity has been performed so far. Generally, LPMOs have been used in conversion experiments and processes under the conditions shown in Table 1. A higher concentration of reductant and higher pH values increased the catalytic turnover. However, both also lead to faster inactivation of LPMO. These observations were attributed to the lower redox potential of the reductant, which better fuels LPMO but also generates more reactive oxygen species that deactivate the LPMO. Therefore, the pH most suitable to test LPMO activity was suggested in the slightly acidic range of around 6.0. 

The role of LPMO inhibitors is only discussed very little in the literature. To date, it has been shown that fermented persimmon juice and tannic acid inhibit LPMO activity [20], but the molecular determinants of this inhibition remain to be investigated. Tokin et al. also identified cinnamtannin B1 as a specific inhibitor of LPMO with a LC_50_ of 0.46 ± 0.04 mM [21]. A crystal structure with a bound inhibitor showed that the molecule bound close to the active site of the enzyme, thus potentially blocking the interaction with substrates or reducing agents. In other literature, inhibition has been mainly discussed for EDTA as a chelating agent, which partially or fully removes the copper from the active site [3,14,22], laccases as O_2_ competing enzymes [23], peroxidases or catalases as H_2_O_2_ competing enzymes [7,24] and cyanide as a copper-binding analog to oxygen or H_2_O_2_ [3,22,25]. A structural study of *Ls*LPMO9A (pdb: 5acj) shows two water molecules ligating the copper in the axial and equatorial position in a crystal structure without a bound substrate. However, when cellotriose is bound to LPMO, the axial water molecule is displaced by the substrate and the equatorial water is replaced by a chloride ion [15]. Forsberg et al. reported the crystal structure of *Sc*LPMO10B (pdb: 4oy6) that shows an acetate molecule bound to the copper, ligating the copper in the axial and equatorial position. The fact that small molecules could easily chelate LPMO is obvious, considering the solvent-exposed active site. 

In this study, we aimed to gain insight into the molecular determinants of LPMO inhibition by carboxylic acids. We used a turbidimetric assay to investigate LPMO’s peroxygenase activity and used the H_2_O_2_-based photometric 2,6-DMP assay and the hydrocoerulignone assay to identify potential LPMO inhibitors. Molecules and ions present during fungal growth on biomass or in cultivation media and related compounds were tested for their capacity to inhibit LPMO activity. Identified inhibitors were then tested again by HPLC and the turbidimetric assay for their ability to reduce the LPMO depolymerization rate of PASC. Finally, several inhibitors were studied by QM simulations to investigate the type and strength of their interaction with LPMO.

## 2. Materials and Methods

### 2.1. Materials and Enzymes

All chemicals were of the highest purity grade available and were purchased from Sigma-Aldrich unless stated otherwise. Hydrocoerulignone was obtained from MP Biomedicals. The *Neurospora crassa* lytic polysaccharide monooxygenase (*Nc*LPMO9C, accession number EAA36362.1) and cellobiose dehydrogenase (*Nc*CDHIIA, accession number XP_956591.1) were recombinantly expressed in *Pichia pastoris* X-33 as previously described [26,27]⁠. Production in a 5-L bioreactor and column purification were also performed according to this publication. The purity of the enzymes was verified by SDS-PAGE.

### 2.2. Determination of LPMO and H_2_O_2_ Concentration

A 0.3 mm quartz cuvette was used to determine the enzyme concentration for *Nc*LPMO9C at 280 nm using the molar-absorption coefficient ε_280_ = 46,910 M^−1^ cm^−1^ and a molecular mass of 34,300 g mol^−1^, and *Nc*CDHIIA was measured at 420 nm using the molar-absorption coefficient ε_420_ = 100,000 M^−1^ cm^−1^ and a molecular mass of 83,587 g mol^−1^. For H_2_O_2,_ a 10 mm quartz cuvette and the absorption at 240 nm with the molar absorption coefficient of ε_240_ = 43.6 M^−1^ cm^−1^ was used to determine its concentration.

### 2.3. LPMO Turbidity Assay

LPMO activity was measured by the attenuation of optical density at 620 nm at 30 °C in 100 mM sodium acetate, 100 mM sodium citrate, or 100 mM sodium phosphate buffer, pH 6.0. For oxalic acid inhibition, different ratios between acetic acid and oxalic acid concentrations were applied to keep the molarity constant. The pH was checked before and after the measurements. A total volume of 2.5 mL contained 100 mM of buffer, pH 6.0, 0.8 mg/mL PASC, 0.5 or 1.0 µM of *Nc*LPMO9C, 1 mM ascorbic acid or 1 µM *Nc*CDHIIA with 10 mM lactose. A 10 mm quartz cuvette with a magnetic stirrer was used to maintain a homogeneously mixed PASC suspension.

### 2.4. LPMO-Activity Assays Based on 2,6-DMP or Hydrocoerulignone

LPMO-activity assay using 2000 µM 2,6-DMP or 500 µM hydrocoerulignone were carried out in 100 mM sodium acetate buffer at pH 6.0, at 30 °C using 100 µM H_2_O_2_, at 469 nm and a reaction time of 300 s. For blank reactions, the same experiments were performed without adding LPMO. The resulting rate was subtracted from the rate with the addition of LPMO. One unit of LPMO activity is defined as the conversion of either 2 µmol 2,6-DMP or 1 µmol hydrocoerulignone or the formation of 1 µmol coerulignone (ε_469_ = 53,200 M^−1^ cm^−1^) per min under reaction conditions. For the inhibition tests, the 100 mM sodium acetate buffer at pH 6.0 was supplemented with 100 mM of the different compounds and the pH was checked before and after the measurements.

### 2.5. PASC Batch-Conversion Experiments

*Nc*LPMO9C (1 µM) was incubated with 2 mg mL^−1^ PASC, either 1 mM ascorbic acid or 1 µM *Nc*CDHIIA together with 10 mM lactose as reductant and 400 µM H_2_O_2_ (4 × 100 µM) as cosubstrate at a total volume of 1 mL. At the beginning and after every hour, 100 µM H_2_O_2_ was added to the reaction. The reaction tubes were closed and continuously mixed in the dark for a total reaction time of 4 h. Reactions were stopped by heating to 95 °C for 10 min. The remaining PASC was removed by centrifugation, and the supernatant was analyzed by HPLC. Soluble cello-oligomers (DP 1–6) were used as standards to quantify LPMO-released products. To test the effect of different buffers or inhibitors, 100 mM sodium acetate buffer, pH 6.0, was supplemented with 100 mM of the different compounds.

### 2.6. MD Simulations

The initial structure was taken from the PDB database with the pdb entry: 4d7u. The structure was parametrized using the GROMOS++ software package [28] with the GROMOS 54A7 force field [29]. Partial atomic charges around the copper ion of LPMO were obtained from density functional theory (DFT) calculations on the Cu(II) center and its coordination ligands, as described previously [30]. Afterwards, the GROMOS topology was converted to the GROMACS topology format. All simulations were performed with the GROMACS 2018.2 software package. The protein was solvated in a periodic rectangular simulation box containing the simple point-charge water model [31] with a minimal solute-wall distance of 1 nm. Chloride and sodium counterions were added to create an overall neutral system at pH 7.0. The structure was relaxed by an in vacuo steepest-descent energy minimization with a convergence criterion of 0.1 kJ mol^−1^. Afterwards, the system was heated to 298 K over 0.1 ns and equilibrated at 1 bar using the Berendsen barostat. For all MD simulations, a leapfrog integration scheme with a time-step of 2 fs was used, and covalent bonds were constrained to a constant distance using the LINCS algorithm. A group-based cut-off scheme for neighbor searching was performed using every 5 steps with a cut-off sphere of 1.4 nm. Nonbonded electrostatic and Lennard-Jones interactions were calculated within a cut-off sphere of 1.4 nm. Electrostatic interactions were calculated by a reaction-field contribution with a relative dielectric permittivity of 61 beyond the cut-off sphere.

### 2.7. QM Calculations

An initial active site model was constructed from *Nc*LPMO9C (pdb entry: 4d7u) using residues within 5 Å of the Cu(II) with the two coordinated water molecules. The considered model thus contains the copper atom, 3M-His1, Thr2, His83, His155, Gln164, Tyr166 and two coordinated water molecules. All C- and N-terminal groups were replaced by hydrogen atoms to saturate the valence shell of the Cα atom. In further calculations, positions of Cα and Cβ were fixed in their initial positions to approximate the structure of the enzyme active site. The rest of the atoms remained without any restraints. The structure was optimized using the density functional theory approach with B3LYP functional [32,33] and 6–31G(d) basis set [34,35]. All calculations were performed in Gaussian 09 software package [36]. The SCF energy cut-off was 3 × 10^−5^ kJ mol^−1^, and the final RMS energy gradient was below 1.5 kJ mol^−1^ Å^−1^. For open-shell systems, the unrestricted Kohn–Sham formalism was applied. The observed spin contamination was up to 0.010. Vibrational frequency analysis proved the final minimized geometries to be real minima with no imaginary frequencies. Total electronic energies were refined using a larger 6-311++G(d,p) basis set [37]. The effect of water and the protein environment was included using the integral equation formalism variant of the polarizable continuum model (IEFPCM) as implemented in Gaussian 09 [36]. To simulate the protein environment, diethyl ether (DEE) parameters with ε = 4.2 were used.

Structures of inhibitory complexes were created by replacing the water molecules with the ligand. For some ligands, more than one binding pose is possible either through a different functional group, a different orientation or a different position. All relevant binding poses were calculated, and only the most relevant complexes, i.e., those with the lowest total electronic energies, were picked for further study.

The total binding energy (Δ*E*_bind_) of a ligand (**L**) to the active site (**A**) was calculated as

$$\Delta E_{\mathrm{bind}}\left(AL\right)=E\left(AL_{\left(\mathrm{DEE}\right)}\right)-E\left(A_{\left(\mathrm{DEE}\right)}\right)-E\left(L_{\left(H_{2}O\right)}\right)+\Delta E_{\mathrm{BSSE}}$$

where *E*(**X**) is the total electronic energies of corresponding species in their optimized geometry in the specified solvent and Δ*E*_BSSE_ is the basis set superposition error (BSSE). To calculate BSSE, counterpoise correction of Boys and Bernardi was used [38]:

$$\Delta E_{\mathrm{BSSE}}=\Delta E_{\mathrm{BSSE}}\left(A\right)+\Delta E_{\mathrm{BSSE}}\left(L\right)$$

$$\Delta E_{\mathrm{BSSE}}\left(A\right)=E^{\mathrm{AL}}\left(A\right)-E^{A}\left(A\right)$$

$$\Delta E_{\mathrm{BSSE}}\left(L\right)=E^{\mathrm{AL}}\left(L\right)-E^{L}\left(L\right)$$

where Δ*E*_BSSE_(**A**) and Δ*E*_BSSE_(**L**) are estimates of artificial stabilization due to basis functions from the binding partner. They can be expressed as a difference between the total electronic energy of the monomer with basis sets of the dimer (*E*_AL_(**A**) and *E*_AL_(**L**)) and the total electronic energy of the monomer with its own basis sets (*E*_A_(**A**) and *E*_L_(**L**)).

The total binding energy can be equivalently expressed as a sum of the contributions from partial steps:

$$\Delta E_{\mathrm{bind}}\left(AL\right)=\Delta E_{\mathrm{int}}+\Delta E_{\mathrm{def}}\left(A\right)+\Delta E_{\mathrm{def}}\left(L\right)+\Delta E_{\mathrm{dehydr}}\left(L\right)+\Delta E_{\mathrm{BSSE}}$$

where Δ*E*_int_ is the interaction energy, Δ*E*_def_(**A**) and Δ*E*_def_(**L**) are deformation energies of the active site and the inhibitor, respectively, due to their interaction, Δ*E*_dehydr_(**L**) is the dehydration energy of the inhibitor, and *E*_BSSE_ is the basis set superposition error. These contributions can be then calculated as:

$$\Delta E_{\mathrm{int}}\left(AL\right)=E\left(AL_{\left(\mathrm{DEE}\right)}\right)-E\left(A_{\mathrm{AL},\;\left(\mathrm{DEE}\right)}\right)-E\left(L_{\mathrm{AL},\;\left(\mathrm{DEE}\right)}\right)$$

$$\Delta E_{\mathrm{def}}\left(A\right)=E\left(A_{\mathrm{AL},\;\left(\mathrm{DEE}\right)}\right)-E\left(A_{\left(\mathrm{DEE}\right)}\right)$$

$$\Delta E_{\mathrm{def}}\left(L\right)=E\left(L_{\mathrm{AL},\;\left(\mathrm{DEE}\right)}\right)-E\left(L_{\left(\mathrm{DEE}\right)}\right)$$

$$\Delta E_{\mathrm{dehydr}}\left(L\right)=E\left(L_{\left(\mathrm{DEE}\right)}\right)-E\left(L_{\left(H_{2}O\right)}\right)$$

where *E*(**A**_AL,(DEE)_) and *E*(**L**_AL,(DEE)_) are total electronic energies of monomer **A** and **L**, respectively, in the optimized geometry of the dimer in DEE, *E*(**A**_(DEE)_) and *E*(**L**_(DEE)_) are the energies in the optimized conformations of the monomers in DEE and $E\left(L_{\left(H_{2}O\right)}\right)$ is in water.

Thermal correction to the free energy of binding can be obtained as an algebraic sum from the thermal corrections of the complex and monomers:

$$\Delta G_{\mathrm{bind},\mathrm{corr}}\left(AL\right)=\Delta G_{\mathrm{corr}}\left(AL_{\left(\mathrm{DEE}\right)}\right)-\Delta G_{\mathrm{corr}}\left(A_{\left(\mathrm{DEE}\right)}\right)-\Delta G_{\mathrm{corr}}\left(L_{\left(H_{2}O\right)}\right)$$

Values of the thermal corrections to free energy of the molecule, Δ*G*_corr_(**X**), were calculated from the translational, rotational and vibrational partition functions obtained from the vibrational analysis within the rigid-rotor/harmonic-oscillator approximation in 6-31G(d) basis set at 298.15 K.

The binding free energy is then:

$$\Delta G_{\mathrm{bind}}\left(AL\right)=\Delta E_{\mathrm{bind}}\left(AL\right)+\Delta G_{\mathrm{bind},\mathrm{corr}}\left(AL\right)$$

Assuming the competitive inhibition and classical Michaelis–Menten kinetics, the inhibitor binding affinity *K_i_* is related to the IC_50_ values by the equation proposed by Cheng and Prusoff [39]:

$$K_{i}=\frac{{\mathrm{IC}}_{50}}{1+\frac{\left[S\right]}{K_{M}}}$$

where [S] is a substrate concentration, and *K_M_* is the Michaelis constant. The dissociation constant of a complex *K_D_* can be expressed from binding free energy as

$$K_{D}=e^{-\frac{\Delta G_{\mathrm{bind}}}{RT}}\;$$

We can roughly assume that

$$K_{i}\approx \frac{1}{K_{D}}$$

The LPMO reaction and inhibition mechanisms are rather complex with many uncertainties, while multiple steps are expected to be inhibited similarly. Therefore, the relationship between the observed IC_50_ values and the calculated Δ*G*_bind_ cannot be analytically derived. Nevertheless, we can still expect a linear relationship between the logarithm of IC_50_ and Δ*G*_bind_:

$$\ln\left({\mathrm{IC}}_{50}\right)\approx a+b\Delta G_{\mathrm{bind}}$$

## 3. Results

### 3.1. Assessing the Peroxygenase Reactivity of LPMO

**Figure 1:**
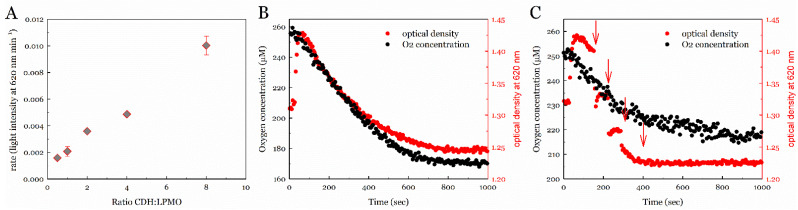
Decrease in attenuation in the turbidimetric assay of PASC depolymerization at pH 6.0 and 30 °C. (**A**) Varying ratios of CDH in regard to 2 µM LPMO show a linear correlation of the obtained LPMO activities. (**B**) The decrease in optical density and oxygen concentration correlate and indicate that oxygen is directly or indirectly consumed during the reaction. (**C**) Repeated addition of 20 µM H_2_O_2_ (red arrows) demonstrates that the fast degradation of PASC is correlated with the addition/production of H_2_O_2_.

We used a turbidimetric assay to determine the peroxygenase reactivity of *Neurospora crassa* LPMO9C (LPMO) in the presence of phosphoric-acid-swollen cellulose (PASC) [40]. Because PASC is insoluble in an aqueous solution, it scatters light passing the solution. As a result of LPMO cleaving glycosidic bonds and releasing soluble cello-oligosaccharides, the light attenuation decreases over time [40,41]. The reaction setup consisted of a stirred cuvette tempered to 30 °C, to which 3 µM LPMO and 2 µM *Neurospora crassa* cellobiose dehydrogenase IIA (CDH) as the native reductant and source of H_2_O_2_ were added. The reaction had a total volume of 2.5 mL and contained 10 mM cellobiose as a substrate for the CDH. The concentration of LPMO was kept constant at 1 µM, while different amounts of CDH (from 1 µM to 16 µM) were added to the assay (Figure 1A). The activity of LPMO increased linearly with increasing CDH concentration, which suggests a fast electron transfer between CDH and LPMO that is substantially faster than the steady-state catalytic rate of LPMO. To assess the O_2_ consumption during the reaction, an oxygen microsensor was placed in the measurement cuvette, allowing the simultaneous measurement of the dissolved oxygen and the optical density (Figure 1B). In CDH-driven assays using 1 µM LPMO and 2 µM CDH, the dissolved-oxygen concentration and the optical density decreased at a comparable rate, suggesting that the oxygen depletion in the reaction system was connected to substrate degradation by LPMO. The oxygen consumption by 2 µM CDH (5.3 µmol^−1^ min^−1^ mL^−1^) was lower than in combination with LPMO and PASC (13.0 µmol^−1^ min^−1^ mL^−1^), which indicates that also LPMO produces H_2_O_2_ in an uncoupling reaction. In the absence of PASC (CDH and LPMO), the oxygen consumption showed a biphasic behavior with a fast initial rate (for ca. 3 min, 17.6 µmol^−1^ min^−1^ mL^−1^) and a slower second rate (3.4 µmol^−1^ min^−1^ mL^−1^), reflecting the instability of LPMO in the absence of substrate (Figure S1). When higher concentrations of H_2_O_2_ were added externally at aliquots of 20 µM (Figure 1C), the oxygen-consumption rate remained the same, while the optical density decreased within the mixing time of the instrument (~10 s). We observed rapid degradation of the PASC, which demonstrates that the H_2_O_2_ produced by CDH and the uncoupling reaction of LPMO is consumed by LPMO. However, these reactions also demonstrate fast deactivation of the LPMO. Upon adding H_2_O_2_, the LPMO reaction also rapidly diminished after 400 s and the decrease in optical density ceased after 600–800 s.

### 3.2. Screening for LPMO Inhibitors

**Figure 2:**
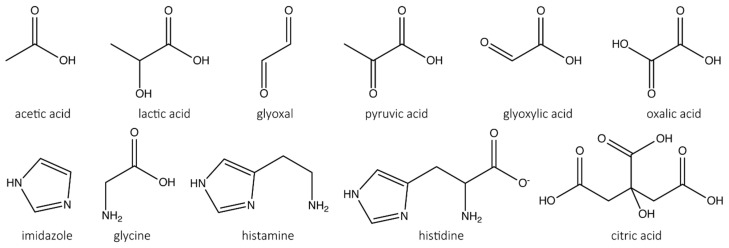
Mono-, di-, and tricarboxylic acids and amino acids or derivatives employed in the screening assay.

To screen for reaction conditions that improve the stability of LPMO, we used two photometric assays, the 2,6-DMP and the hydrocoerulignone assay, to measure the activity of *Nc*LPMO9C in the absence or presence of a range of carboxylic acids (Figure 2). 

Analogs of oxalic acid and histidine were tested and showed a decrease in specific activities in the following order: acetic acid > lactic acid > glyoxal > pyruvic acid > glyoxylic acid > oxalic acid for oxalic acid analogs and imidazole > glycine > histamine > histidine for histidine analogs (Table 2). Furthermore, citric acid as a tridentate species also inhibited the LPMO activity comparable to oxalic acid. The more sensitive assay based on hydrocoerulignone shows a similar effect as the 2,6-DMP assay. The residual activities of lactic acid or glyoxal are twice as high for the hydrocoerulignone assay than for the 2,6-DMP assay. For histidine analogues, this effect was not observed; a different inhibition mechanism could be effective. Higher concentrations (>100 mM) of oxalic acid, histidine or citric acid decreased the peroxidase activity of LPMO to a nondetectable level. 

### 3.3. Determination of the Half-Maximal Inhibitory Concentration (IC_50_)

By measuring the LPMO activity in 100 mM sodium acetate buffer, we determined the maximum activity achievable for the applied conditions. By supplementing the 100 mM sodium acetate buffer with different concentrations of inhibitors, we calculated the inhibitor concentration at 50% activity (IC_50_, Table 3). 

The most potent inhibitory effect was observed for oxalic acid and histidine, with IC_50_ values of around 1 mM. These two compounds are bidentate species known to chelate metal ions. Citric acid as a tridentate species shows similar but not lower IC_50_ values with both assays (Figure 2, Table 3). A similar observation was made for histidine and histamine. Histidine shows a higher inhibitory effect than histamine, which can be concluded from a stabilizing effect of the carboxylic group for chelating the copper active site. Only a minor inhibitory effect could be observed for other amino acids. The IC_50_ values decrease in the following order for other amino acids tested: glycine > asparagine > phenylalanine > histamine > histidine. Fluoride did not show any effect on the activity of the LPMO until very high concentrations were used, which most likely affected the assay and not the LPMO activity. Sulfate shows a weak inhibitory effect, with an IC_50_ value of around 30 mM, which can be concluded as the interaction of the oxyanion. All observations were made for the peroxidase reaction of LPMO; therefore, we tested the most potent inhibitor (oxalic acid) with other LPMO catalyst reactions, such as the H_2_O_2_ uncoupling [26] and the natural oxygenase reaction [7,42].

### 3.4. Inhibitory Effect on H_2_O_2_ Production by LPMO

The Amplex Red–horseradish peroxidase assay was used to measure the uncoupling reaction of *Nc*LPMO9C that produces superoxide or H_2_O_2_. The activity of LPMO was measured in the presence of oxalic acid as an inhibitory compound. The specific activity for *Nc*LPMO9C in 100 mM sodium acetate buffer at pH 6.0 with *Nc*CDHIIA as a reducing agent was determined to be 4.9 ± 0.1 U g^−1^. The addition of 100 mM oxalic acid gave no detectable activity (0.1 ± 0.1 U g^−1^), whereas the H_2_O_2_ production by *Nc*CDHIIA was not inhibited (Table S1). Altogether, oxalic acid seems to inhibit the uncoupling reaction of LPMO, which can be further interpreted as chelating the copper site and avoiding the electron transfer from CDH to LPMO or the binding of oxygen to the active site. It is also unclear whether superoxide is released upon uncoupling, which dismutates to H_2_O_2_, or if H_2_O_2_ is directly released from the active site.

### 3.5. Turbidity Assay for Detecting LPMO Activity on PASC in the Presence of Inhibitors

**Figure 3:**
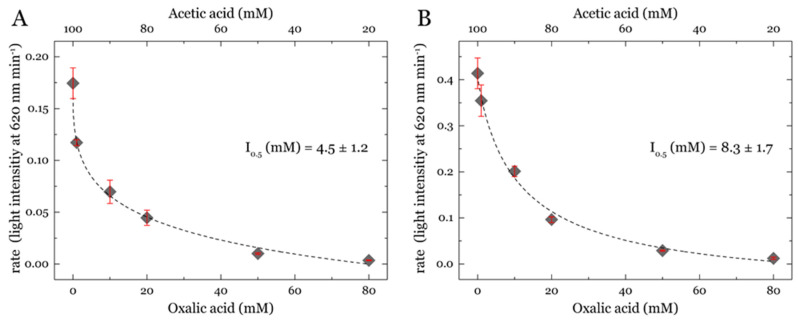
IC_50_ plots showing inhibition of the peroxygenase reaction rate for *Nc*LPMO9C with increasing oxalic acid and decreasing acetic acid concentration. LPMO peroxygenase activity was tested on PASC using a turbidity assay. Reaction was started with H_2_O_2_ and rates were obtained from light-intensity decrease at 620 nm. Assay contained 0.5 µM (**A**) or 1.0 µM (**B**) *Nc*LPMO9C, 0.8 mg mL^−1^ PASC, 1 mM ascorbic acid and 100 µM H_2_O_2_, respectively. H_2_O_2_ was added after 30 s to the stirred cuvette and the rate calculated from 40 s up to 200 s, having a data interval for each point of 0.1 s. pH was checked after all measurements and data are expressed as mean values (±SE), from three independent repeats. IC_50_ values were obtained by a nonlinear curve fit and calculating the oxalic acid concentration of oxalic acid at 50% of the rate. Note that reactions without addition of H_2_O_2_ showed no decrease in light intensity over the measuring time.

After finding that oxalic acid is inhibiting the peroxidase and superoxide uncoupling reaction of LPMO, we further tested the monooxygenase reaction in the presence of oxalic acid as an inhibitor. Therefore, PASC was used as a cellulosic substrate and the LPMO activity was detected by measuring the decrease in turbidity (Figure 3). To measure the inhibition by oxalic acid, the change in light intensity per minute was calculated at different inhibitor concentrations. To avoid concentration effects, we chose different ratios of acetic acid and oxalic acid. LPMO was reduced with ascorbic acid and mixed with H_2_O_2_ to measure the activity. Under the used reaction conditions and measuring time, no change without the addition of H_2_O_2_ was visible. However, titrating H_2_O_2_ to the solution ended in a fast change in light intensity, which can be interpreted as LPMO activity on PASC releasing soluble cello-oligosaccharides. Changing the acetic acid and oxalic acid ratio showed that oxalic acid inhibits the peroxygenase reaction. For reactions with ascorbic acid, the IC_50_ values are around three or six times higher than the IC_50_ values obtained from the peroxidase reaction with 2,6-DMP and hydrocoerulignone. However, they also differ when changing the LPMO concentration. We want to note that for measurements with ascorbic acid, the reaction was so quick that we reached the limit of the assay regarding the signal-to-noise ratio and measurement time for the rate calculation. Consequently, it is likely that the fastest rates obtained with ascorbic acid are undervalued and that the IC_50_ values are lower. We therefore conclude that oxalic acid dramatically inhibits the reaction rate at higher concentrations and directly interacts with the copper center.

Additionally, we measured the rate of PASC degradation in the presence of other inhibitors to compare it to the data observed with the peroxidase assays from above. Table 4 shows the increase in light intensity over time and the calculated residual activity based on the rate observed from 100 mM sodium acetate buffer at pH 6.0. A similar trend was observed compared to the peroxidase reaction. Acetic acid shows minor inhibition if the concentration is doubled (100 to 200 mM). Oxalic acid shows the strongest inhibition, and no change in light intensity over time was observed. The rates decrease in the following order: acetic acid > phosphoric acid > lactic acid > citric acid > oxalic acid. 

### 3.6. Batch Conversion of Cellulose with LPMO and Analysis Using HPLC in the Presence of an Inhibitor

**Figure 4:**
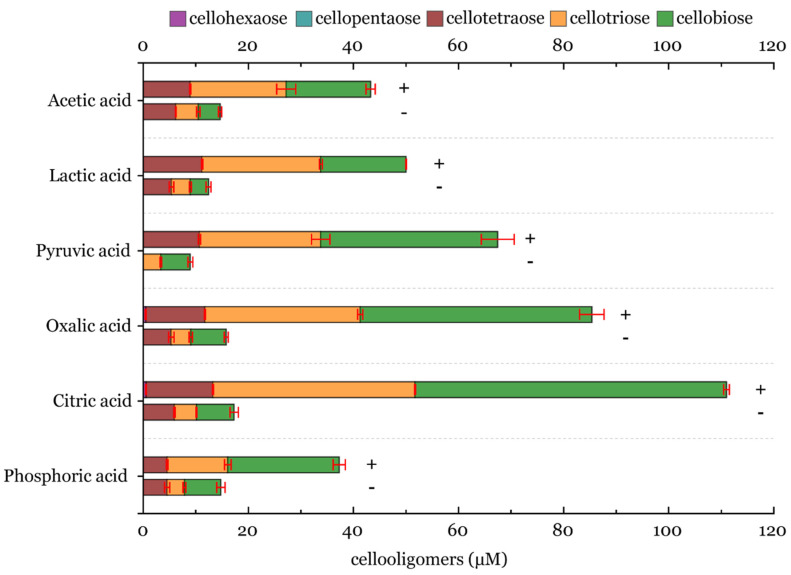
Product release by *Nc*LPMO9C analyzed with HPLC during batch conversions after 4 h in the presence of inhibitors, 1 mM ascorbic acid as reductant and 100 µM H_2_O_2_ as a cosubstrate. H_2_O_2_ was added four times every hour. The graph shows staked bars of the cello-oligosaccharide concentrations with (+) and without (−) reductant.

We analyzed cello-oligomeric product formation after PASC treatment with *Nc*LPMO9C, which is released as a byproduct of cellulose oxidation. Only nonoxidized cello-oligomers were identified by standards, and their concentration was calculated (Figure 4, Table S2). Conditions with different buffers and reducing agents were tested. In all experiments, LPMO, PASC and H_2_O_2_ were present. As a reducing agent, ascorbic acid was chosen, whereas blank reactions were carried out in the absence of ascorbic acid. The highest product release after a total reaction time of 4 h was observed in reactions with citric acid (111 µM cello-oligosaccharides) and oxalic acid (85 µM). LPMO in the presence of lactic and pyruvic acid released around 50 to 67 µM of cello-oligosaccharides, whereas in reactions with acetic and phosphoric acid, similar cello-oligosaccharide concentrations of around 40 µM were released. For reactions without ascorbic acid, 9–17 µM of cello-oligosaccharide products were detected. The production of nonoxidized cello-oligomers increase in the following order by the added inhibitors: phosphoric acid > acetic acid > lactic acid > pyruvic acid > oxalic acid > citric acid (Figure 4, Table S2). *N. crassa* LPMO9C released twice the amount of cello-oligomers in the presence of chelating compounds such as oxalic or citric acid. 

### 3.7. Structural Causes of LPMO Copper Active Site Inhibition

**Figure 5:**
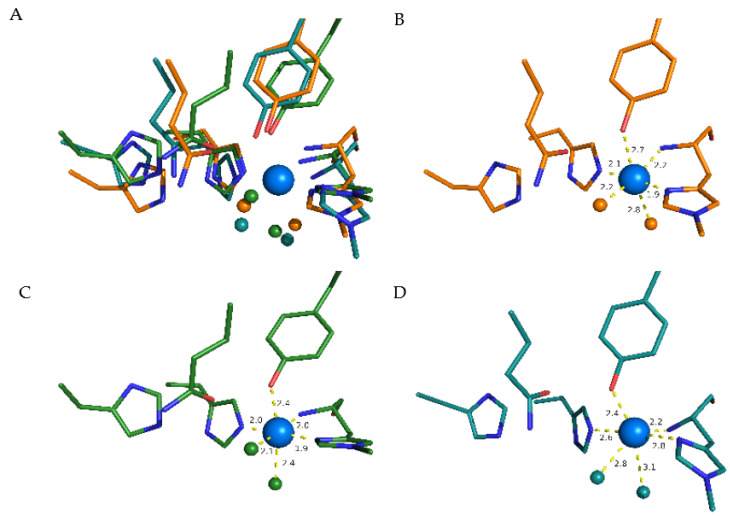
Comparison of active-site geometries regarding water molecules surrounding the copper atom. (**A**) Superimposition of *Ls*LPMO9A (pdb: 5acg; orange), *Nc*LPMO9C (pdb: 4d7u; QM-optimized; green) and *Nc*LPMO9C (clustered from MD Simulation; cyan) structures. (**B**–**D**) show same structures in a single image, including distances. (**B**) Active site from *Ls*LPMO9A (pdb: 5acg) crystal structure (orange), (**C**) *Nc*LPMO9C (pdb: 4d7u) QM-optimized structure (green) and (**D**) clustered structure of *Nc*LPMO9C from a 200 ns MD-Simulation (cyan). Bigger blue sphere represents copper atom and smaller spheres oxygen atoms of water molecules. The hydrogen atoms of all amino acid residues are hidden.

**Figure 6:**
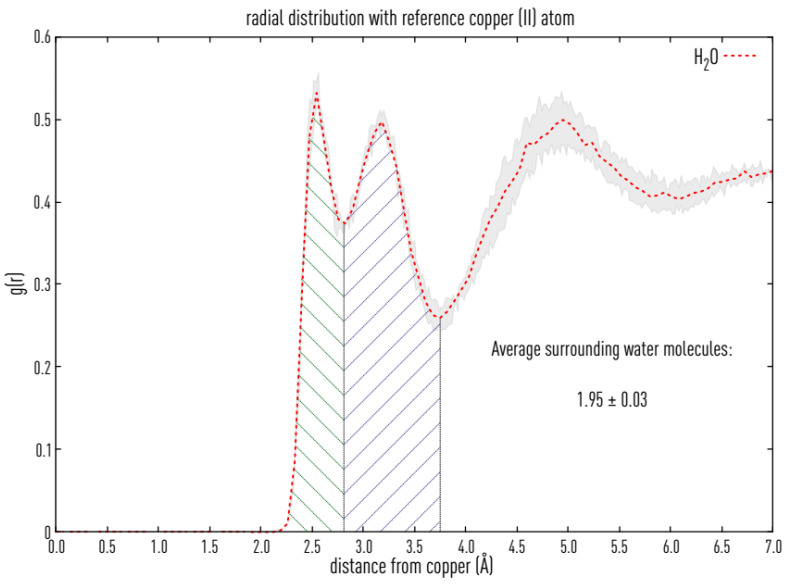
Radial distribution of water molecules with reference copper atom in the active site of *Nc*LPMO9C and the calculated water molecules surrounding the copper atom during 200 ns MD-Simulation. Calculation based on the integration of the marked area until the second minimum. Green and blue dashed areas represent first and second hydration shells of the copper atom. Data are mean values (±SD) of 3 independent simulations.

As a bidentate anionic species, oxalic acid could interact with the copper atom in LPMOs. Therefore, we investigated the active site of LPMOs from the AA family 9. The copper atom in the active site of LPMOs is solvent-exposed [43,44] and is coordinated by three equatorial nitrogen atoms and one axial hydroxyl group. In a typical octahedral or distorted octahedral copper geometry, one equatorial and one axial position is free in solution and occupied by water molecules (Figure 5A). The crystal structure of *Ls*LPMO9A (pdb: 5acg) was resolved at a resolution of 1.9 Å and shows a typical copper coordination with the histidine brace, a tyrosine and two water molecules ligating the copper (Figure 5B). The crystal structure of *Nc*LPMO9C has a resolution of 1.6 Å; however, it lacks water molecules. Therefore, the two water molecules were added to the structure of *Nc*LPMO9C, and subsequent QM optimization (Figure 5C) of the active site residues gave a very similar structure to *Ls*LPMO9A. Furthermore, a clustering (2.5 Å cut-off) of the *Nc*LPMO9C trajectory from a 200 ns simulation showed a main cluster having a similar active site to the crystal structure with two water molecules close to the active site copper (Figure 5D). Calculating the hydration shells around the copper atom over the 200 ns simulation can be used to obtain more information on the copper active site geometry. The water molecules surrounding the copper atom can be determined by solving the radial distribution function (rdf), which is defined as the probability of finding an H_2_O molecule at a given distance from the copper atom relative to the same probability for a homogeneous distribution of water. In Figure 6 the two main hydration shells are visible and show that the copper atom is mainly surrounded by a water molecule within a distance of 2.5 Å and a second water molecule within a distance of 3.2 Å. This is in correspondence with the observed structure from the clustering, that the equatorial water is in closer vicinity compared to the axial water molecule. We conclude that the copper atom can be ligated by bi- or tridentate chelating compounds by replacing the two water molecules. 

However, the structure and properties of the inhibitory complex can be more precisely described using QM calculations. In Figure 5, we already showed that the active-site structure remains preserved after energy minimization. The calculated structural data are summarized in Table S3. In the optimized structure, the Cu(II) is hexacoordinated by five ligands, i.e., (*Nc*LPMO9C) Me-His1 and His83, forming the histidine brace, the phenolic group of Tyr166 and two water molecules. Me-His1 is bidentate ligand binding through N1 of the imidazole ring with a bond length of 1.950 Å and the nitrogen atom of the N-terminal group (2.045 Å). The second histidine binds through the N3 atom of the imidazole group (1.972 Å). The coordinate bond formed between the copper atom and oxygen of the phenolic group of tyrosine is distinctly longer (2.441 Å) as well as with one water molecule (2.340 Å), both in axial positions. The remaining position is occupied by another water molecule. With a bond length of 2.071 Å, it can be considered equatorial, and the shape of the copper coordination sphere is thus a distorted tetragonal bipyramid.

Some insight into the electronic structure can be obtained by the atomic charge and spin-density assignment listed in Table S3. The formal charge of +2 on the copper atom is decreased to around +1.2 by donor–acceptor transfer from ligands. All donor atoms carry a charge between −0.63 and −0.97. The most negative are the oxygens of water and the N-terminal. This is mainly due to the fact that the remaining ligands can effectively distribute their electrons along their aromatic rings. The electron transfer from acceptor to donor is also expressed by the reduced spin density to 0.74 at the copper atom. The rest is distributed mainly between Me-His1 and His83 in an equatorial plane, as shown in Figure S3. 

### 3.8. Inhibitor–Catalytic Site Complexes

We have modeled complexes of the *Nc*LPMO9C active site with several potentially polydentate ligands: citric acid, oxalic acid, histidine, pyruvic acid, glyoxylic acid, histamine, phenylalanine, asparagine and glycine. Table 5 summarizes the calculated structural data. The structures of the complexes are depicted in Figure S4. The binding energies Δ*E*_bind_ are collected in Table S4 expresses the energy released by the complex formation and corresponds to the complex stability. Within this set, the most negative binding energies were observed for citric and oxalic acid. These ligands form two coordinate bonds with copper. The oxalic acid molecule in this complex is planar, with Cu–O bonding distances of 1.978 and 2.146 Å. Moreover, one O-atom of oxalic acid forms a hydrogen bond with the H-atom of the amidic group of Gln164 with the O–H bond length of 1.857 Å. The total binding energy of the oxalic acid is −255 kJ mol^−1^.

The lowest value of −267 kJ mol^−1^ was observed for citric acid. From several tested citrate-Cu(II)-LPMO complexes, the one depicted in Figure S3 is the most stable one, with total electronic energy lower by 35 kJ mol^−1^. This structure is distinctive by the six-membered ring created by the hydroxyl group chelating the copper atom together with one of the terminal carboxylic groups. Interestingly, the bipyramidal structure of the copper atom becomes highly distorted in the distance of the phenolic group, which becomes practically dissociated to the Cu distance of 3.36 Å. This complex is further stabilized by the hydrogen bonds between the nonbound –COO^−^ group and N-atoms of two residues—the amidic group of Gln164 and the imidazole group of His155 with plausible O∙∙∙H distance of 1.83 and 1.87 Å, respectively. The proton of the citric acid hydroxyl group becomes partly shared with the free –COO^–^ group, thus stabilizing another favorable 6-membered ring.

Another special case is histidine, which can bind through N-atoms of the imidazole and amine group, or by O-atoms of carboxyl group. The first pose, forming a bidentate complex through N1-atom of imidazole and amino group, is preferred by 26 kJ mol^−1^. Comparable stability of the monodentate carboxyl-linked complex can be explained by the additional π-stacking interaction with Me-His1. 

Turning to histamine, its binding energy is considerably weaker despite no significant structural difference between the histamine and histidine complex. Nevertheless, the binding energy of histamine is by more than 100 kJ mol^−1^ weaker. This difference is hardly attributable solely to the additional hydrogen bond created between the histidine carboxyl group and the Gln164 amidic group. This exceptionally high stabilization could be partly explained by changes in the electronic structure of the amino group invoked by the carboxyl group through the Cα atom. Additionally, as shown in Table S5, both binding atoms of histidine form slightly stronger bonds with the copper atom than histamine, according to the NBO estimation of 2e-stabilization energy. This trend is also evident for the off-site hydrogen bonds with Gln164.

Pyruvic and glyoxylic acid also form a bidentate complex. Interestingly, monodentate pose of glyoxylic acid (see Figure S4) is comparably stable, which can be attributed to the extra hydrogen bond with His155. The stability of the remaining complexes with phenylalanine, asparagine and glycine is comparable to the aquacomplex. Interestingly, phenylalanine forms a bidentate complex over the carboxyl group and the phenyl π-electron cloud.

**Figure 7:**
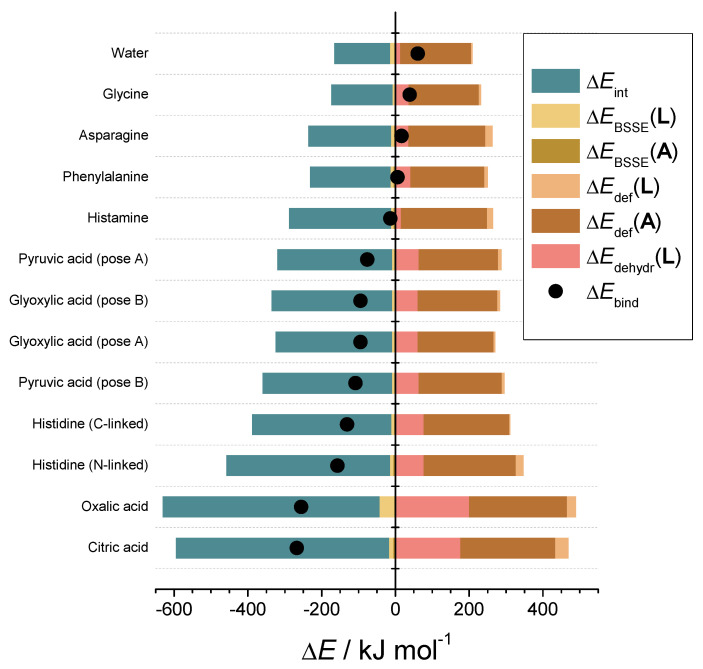
Total binding energies and contributions of interaction, deformation, dehydration and basis set superposition error.

Further analysis of the ligands by their binding free energies Δ*G*_bind_ in Table S4 showed similar trends. As shown in Figure S5, there is a strong correlation between Δ*G*_bind_ and Δ*E*_bind_ with R^2^ = 0.968. The thermal correction to free energy, in fact, only increases differences between various ligands; however, the cost of such calculation is significantly higher. The qualitative results on binding affinity can be already obtained from total electronic energies (Figure 7).

**Figure 8:**
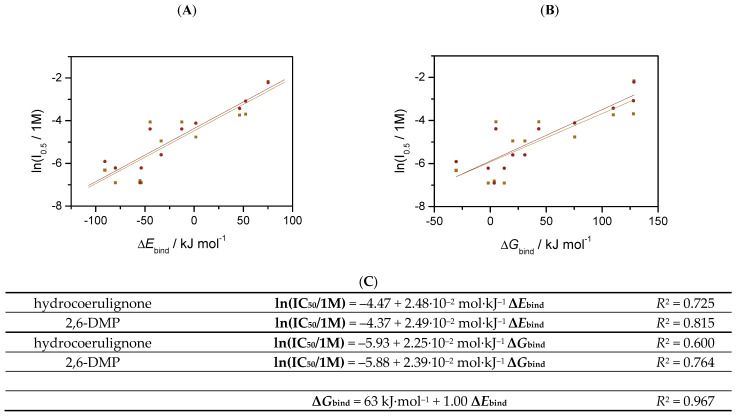
The dependence of the logarithm of IC_50_ on the (**A**) binding energies and (**B**) binding free energies with 2,6-DMP (circles) or hydrocoerulignone (squares) as substrates. (**C**) Equations of the dependence between ln(IC_50_), **Δ*E*_bind_** and **Δ*G*_bind_**.

As for the contributions to binding energy, a similar progression is noticeable for interaction energies. Other contributions are almost constant or very small compared to the interaction energies. The only exception is the dehydration energies, which increase with the charge of the ligand. Finally, we hypothesize that a relationship should exist between the calculated binding energies and the measured IC_50_ values (Table 3). Figure 8 shows a noticeable trend between IC_50_ values and Δ*G*_bind_ (Figure 8A) or Δ*E*_bind_ (Figure 8B). The trend is qualitative, with a decent R-squared of 0.764 and 0.815 (Figure 8C), respectively. Nevertheless, considering the complexity of the LPMO mechanism, this relationship is surprisingly straightforward and explains the observed inhibition on the structural level. This finding potentially allows for the estimation of the inhibitory effects of other small molecules on LPMO.

## 4. Discussion

The efficient reduction of LPMO by CDH was demonstrated in the initially performed turbidimetric assay and supported the reported bimolecular electron-transfer rate from CDH to LPMO (ca 10^6^ M^−1^ s^−1^ [45,46]). This rate is within the same order of magnitude as the reported catalytic efficiency of LPMO’s H_2_O_2_-driven catalysis (ca 10^5–^10^6^ M^−1^ s^−1^ [47]). The electron transfer in the performed turbidimetric reaction was, however, substantially faster than the steady-state catalytic rate of LPMO [42]. The assessment of the O_2_-consumption during the reaction with PASC showed that the dissolved oxygen concentration and the optical density both decreased at a comparable rate, which suggests that both are connected. However, O_2_ is not the direct cosubstrate of LPMO. This has been tested in this study by adding aliquots of 20 µM H_2_O_2,_ during which the oxygen consumption rate remained the same while the optical density decreased quickly. It has been shown previously that the oxidase activity of CDH can support LPMO activity by providing H_2_O_2_ as a cosubstrate for the LPMO reaction [25]. In the absence of PASC, the oxygen consumption showed a biphasic behavior with a fast initial rate and a slower second rate, reflecting the instability of LPMO in the absence of substrate [7]. We observed rapid degradation of the PASC, which is consistent with previous reports showing a fast catalytic reaction of *Nc*LPMO9C with H_2_O_2_ [40,45,48]. These assays demonstrate that the H_2_O_2_ produced by CDH and the uncoupling reaction of LPMO is consumed by LPMO. However, these reactions also demonstrate fast deactivation of the LPMO. 

The screening of oxalic acid and histidine together with structurally related molecules showed that pyruvic acid, glyoxylic acid, citric acid and oxalic acid as well as histamine and histidine inhibit LPMO peroxidase activity with the 2,6-DMP or hyrocoerulignone assay. The most potent inhibitory effect was observed for oxalic acid or histidine, with IC_50_ values of around 1 mM. These two compounds are bidentate species known to chelate metal ions. Citric acid as tridentate species has a similar but not lower IC_50_ value. The reason for this could be that orientation around the active-site copper atom of LPMO is not suited to the same extent for all kinds of chelators. Glyoxylic acid and pyruvic acid, which lack the second carboxylic group present in oxalic acid, are not as potent inhibitors. Glyoxylic acid has an aldehyde functionality, and in the presence of water quickly forms a geminal diol, which is in equilibrium with its hemiacetal dimer form [49,50,51]. Pyruvic acid has a ketone functional group dissociating to its hemiketal form [52] ⁠in aqueous solutions. Glyoxylic acid shows a higher inhibiting effect than pyruvic acid, which can be interpreted as an increased sterical hindrance for pyruvic acid and its hydrated form, finding a possible chelating position around the copper atom due to the methyl group. Similar observations were made for histidine and histamine. Histidine shows a higher inhibitory effect than histamine, which can be concluded from a stabilizing effect of the carboxylic group for chelating the copper active site. Imidazole shows less inhibition than histamine because the additional nitrogen of the latter species could lead to a more stable association and chelating position, which is further increased by the carboxylic group in histidine. Only a minor inhibitory effect could be observed for other amino acids [14]. Thus, we conclude that histidine chelates the copper active site with the amine group nitrogen and the “near” nitrogen Nδ of the imidazole ring, such as the N-terminal histidine in the active site. Fluoride did not show any effect on the activity of the LPMO until very high concentrations were used, which most likely affected the assay and not the LPMO activity. Sulfate shows a weak inhibitory effect with an IC_50_ value of around 30 mM, which can be concluded as the interaction of the oxyanion as already described [14]. 

Since these observations were made for the peroxidase reaction of LPMO, the effects also have to be tested for the H_2_O_2_ uncoupling reaction [26]⁠ and the natural peroxidase reaction [3,7]. The Amplex Red–horseradish peroxidase assay, which is used to quantify the oxygen-dependent uncoupling reaction of LPMO, was inhibited in the presence of oxalic acid. This may be due to the chelation of the copper site by oxalic acid, thus preventing the electron transfer from CDH to LPMO or the binding of oxygen to the active site. In turbidimetric experiments, the replacement of acetic acid in the buffer by oxalic acid ratio led to increased inhibition of the peroxygenase reaction. We therefore conclude that oxalic acid strongly inhibits the reaction rate at higher concentrations and directly interacts with the copper center. The analysis of cello-oligomeric product formation after PASC treatment with LPMO showed, however, that the highest product release afterwards was observed in reactions with citric acid or oxalic acid followed by lactic or pyruvic, and the lowest concentrations in reactions with acetic and phosphoric acid. It has to be stated that only nonoxidized cello-oligomers could be quantified. This increased release of nonoxidized cello-oligomers is inverse to the inhibition observed in the photometric assays and is unexpected. Since we could not determine the concentration of oxidized cello-oligomers, it is unclear whether the total amount of products increased or only the amount of nonoxidized products. Since the inhibition slows down the activity of LPMO and the uncoupling reaction, it could also protect the LPMO from self-inactivation and therefore increase the total turnover number. 

The structural causes of LPMO copper active-site inhibition were investigated by studying the solvent-exposed copper in the active site of LPMO [43,44], which is coordinated by three equatorial nitrogen atoms and one axial hydroxyl group. In a typical octahedral or distorted octahedral copper geometry, one equatorial and one axial position is free in solution and occupied by water molecules. An MD simulation of the hydration shells around the copper atom showed that the copper atom is mainly surrounded by a water molecule within a distance of 2.5 Å and a second water molecule within a distance of 3.2 Å. This is in agreement with the observed structure from the clustering that the equatorial water is in closer vicinity compared to the axial water molecule. We conclude that the copper atom can be ligated by bi- or tridentate chelating compounds by replacing the two water molecules. An in-depth investigation by using QM calculations resulted in one water molecule in the axial position (2.340 Å), and the remaining position is occupied by another water molecule. With a bond length of 2.071 Å, it can be considered equatorial, and the shape of the copper coordination sphere is thus a distorted tetragonal bipyramid. Complexes of the *Nc*LPMO9C active site with several potentially polydentate ligands were used to calculate their binding energies Δ*E*_bind_ [44]. Within this set, the most negative binding energies Δ*G*_bind_ were observed for citric and oxalic acid. Further analysis of the ligands by their binding free energies showed a strong correlation between Δ*G*_bind_ and Δ*E*_bind_.

## 5. Conclusions

Small organic molecules can inhibit both the peroxidase and peroxygenase reaction of LPMO. In addition, the uncoupling reaction of LPMO releasing H_2_O_2_ was also suppressed by the inhibitors. Oxalic acid and citric acid show the strongest inhibitory effects. Our data from simulations and QM calculations show that the most likely inhibition is a competition between the inhibitor and the reducing agent in the oxidized Cu(II) state. A similar trend between the complexation energy and the IC_50_ values could be obtained, which supports the hypothesis that small molecules with dentate properties, such as oxalic acid, are able to interact with the copper in the active site of LPMOs. Furthermore, we recognized that the stability of the used reductants is increased by the addition of chelating molecules. We conclude that in addition to the chelation of LPMO, other trace elements, such as metal compounds, are complexed and cannot interact with the reducing agent. This diminishes the H_2_O_2_ production by ascorbic acid in solution and could stabilize LPMO during catalysis by resulting in lower catalytic rates. This study also shows that LPMO stability can be substantially increased by optimizing the reaction conditions. If fungi use oxalic acid as an effector to modulate LPMO activity and stability needs to be further investigated by physiological studies, this study provides only initial evidence on possible metabolic regulation of LPMO.

## Figures and Tables

**Table 1:** Commonly used conditions for lytic polysaccharide monooxygenases (LPMO) in conversion experiments of cellulose and hemicellulose.

Enzymes ^a^	Ascorbic Acid (mM)	Substrate ^b^	Cosubstrate ^c^	Buffer Species	Conc. (mM)	pH	Temp. (°C)	Reference
*Ls*AA9A	4	PASC, cellooligomers	O_2_	ammonium-formate	100	6.0	20	[15]
*Nc*AA9C-CBM1	1	XG, PASC, cellooligomers	O_2_	sodium-acetate	10	6.0	40	[16]
	1	PASC	O_2_	potassium-phosphate	50	6.0	30	[12]
	2	PASC	O_2_	ammonium-acetate	5	6.0	50	[17]
*Mt*AA9B, D	1	RAC	O_2_	ammonium-acetate	50	5.0	50	[10]
				sodium-citrate/phosphate	50	3.0–8.0	40–50	
*Gt*AA9B	1	PASC	O_2_	Bis-/Tris-HCl	50	6–9	45	[11]
			H_2_O_2_	Bis-Tris-HCl		6.5		
*Sm*AA10A	0.2–5	β-chitin	O_2_	Tris-HCl	20	8.0	37	[3]
	0.1	CNW	H_2_O_2_	sodium-acetate	50	6.1	25	[6]
*Sc*AA10C	1	Avicel	H_2_O_2_	sodium-phosphate	50	7.0	40–50	[7]
*Vc*AA10B-X-Y-CBM73	1	β-chitin nanofibers	O_2_	Bis-Tris-HCl	50	6.8	37	[18]
Cellic CTec2	0.1–10	Avicel	H_2_O_2_	sodium-acetate	50	5.0	50	[19]

^a^ LPMOs from the organisms: *Ls, Lentinus similis; Nc, Neurospora crassa; Mt, Myceliophthora thermophila; Gt, Gloeophyllum trabeum; Sm, Serratia marcescens; Sc, Streptomyces coelicolor; Vc, Vibrio cholera*; Cellic CTec2, enzyme cocktail from Novozymes containing cellulases and LPMOs. ^b^ PASC, phosphoric-acid-swollen cellulose; XG, xyloglycan; RAC, regenerated amorphous cellulose; CNW, chitin nanowhiskers; Avicel, microcrystalline cellulose. ^c^ Atmospheric oxygen or additionally added H_2_O_2_.

**Table 2:** Comparison of specific and residual activities of *Nc*LPMO9C.

	Hydro-Coerulignone	2,6-DMP	Hydro-Coerulignone	2,6-DMP
	Specific activity with 100 mM inhibitor (U g^−1^)		Residual activity to 100 mM acetate buffer (%)	
**Acetic acid**	97.4 ± 7.2	17.5 ± 1.5	70.8 ± 5.2	76.5 ± 6.6
**Lactic acid**	67.6 ± 10.2	6.4 ± 0.3	49.2 ± 7.4	28.1 ± 1.3
**Glyoxal**	60.5 ± 4.9	4.8 ± 0.3	43.9 ± 3.6	20.8 ± 1.5
**Pyruvic acid**	5.6 ± 0.6	0.1 ± 0.1	4.1 ± 0.4	0.1 ± 0.1
**Glyoxylic acid**	1.5 ± 0.4	0.1 ± 0.1	1.1 ± 0.3	0.1 ± 0.1
**Oxalic acid**	0.8 ± 0.2	0.1 ± 0.1	0.6 ± 0.1	0.1 ± 0.1
**Citric acid**	2.0 ± 0.5	0.3 ± 0.1	1.5 ± 0.4	1.3 ± 0.4
**Imidazole**	99.4 ± 5.4	18.3 ± 1.1	72.2 ± 3.9	80.0 ± 4.8
**Glycine**	67.7 ± 1.6	10.1 ± 0.8	49.2 ± 1.2	44.1 ± 3.5
**Histamine**	2.2 ± 0.6	0.8 ± 0.3	1.6 ± 0.4	3.5 ± 1.3
**Histidine**	0.5 ± 0.3	0.1 ± 0.1	0.4 ± 0.2	0.1 ± 0.1

Data were obtained by measuring the peroxidase activity using the 2,6-DMP (2000 µM; LPMO 2 µM) and the hydrocoerulignone (500 µM, LPMO 0.3 µM)-based LPMO-activity assays using 100 µM H_2_O_2_ as cosubstrate at pH 6.0 and 30 °C. Specific activities were calculated for an inhibitor concentration of 100 mM. To retain enough buffer capacity at pH 6.0, 100 mM sodium acetate buffer was supplemented with 100 mM of the nonbuffering substances (lactic acid, glyoxal, pyruvic acid, glyoxylic acid, oxalic acid, glycine, histamine). For buffering substances (acetic acid, citric acid, imidazole, histidine), the specific concentrations are given for 200 mM. Residual activities are based on the specific activity of *Nc*LPMO9C obtained for 100 mM sodium acetate buffer (hydrocoerulignone = 138 ± 12 U g^−1^, 2,6-DMP = 23 ± 1 U g^−1^). Data expressed as mean values (±SD) from at least three independent repeats.

**Table 3:** IC_50_ values (inhibitor concentration at 50% residual activity).

	Hydrocoerulignone	2,6-DMP
	IC_50_ (mM)	
**Oxalic acid**	1.1 ± 0.1	1.0 ± 0.1
**Glyoxylic acid**	7.1 ± 0.2	3.7 ± 0.6
**Pyruvic acid**	17.2 ± 1.0	12.4 ± 0.8
**Citric acid**	1.8 ± 0.3	2.7 ± 0.2
**Histidine**	1.0 ± 0.1	2.0 ± 0.1
**Histamine**	8.5 ± 0.4	16.3 ± 0.7
**Glycine**	115 ± 20	109 ± 6
**Asparagine**	24.9 ± 0.2	45.8 ± 2.8
**Phenylalanine**	23.8 ± 1.1	32.4 ± 3.7
**Na_2_SO_4_**	31.2 ± 4.7	32.8 ± 3.2
**NaF**	142 ± 39	316 ± 17

Assay conditions: 2000 µM 2,6-DMP or 500 µM hydrocoerulignone, 2.0 µM or 0.3 µM *Nc*LPMO9C, 100 µM H_2_O_2_, 100 mM sodium acetate buffer, pH 6.0, 30 °C. The buffer was supplemented with the different inhibitors. Data are expressed as mean values (±SE) from at least three independent repeats. Activities were plotted using SigmaPlot v. 12.5 (Systat Software, San Jose, CA), and the IC_50_ values were calculated by an exponential curve fit.

**Table 4:** Rates obtained for *Nc*LPMO9C by the turbidity assay in the presence of different inhibitors.

	Rate (Light Intensity at 620 nm min^−1^)	Residual Activity (%)
**Acetic acid**	0.14 ± 0.01	84 ± 5
**Phosphoric acid**	0.107 ± 0.02	65 ± 10
**Lactic acid**	0.08 ± 0.02	47 ± 13
**Citric acid**	0.014 ± 0.001	8 ± 1
**Oxalic acid**	0.002 ± 0.001	1.4 ± 0.6

Residual activity is calculated based on the rate (0.17 ± 0.02) observed for 100 mM sodium acetate buffer at pH 6.0 with 0.5 µM *Nc*LPMO9C, 1 mM ascorbic acid and 100 µM H_2_O_2_. All data are expressed as mean values (±SD), from three independent repeats.

**Table 5:** DFT/B3LYP/6-31G(d)-optimized Cu(II)–L bond lengths in Å.

	N_im(Me-His1)_	N_am(Me-His1)_	N_im(His83)_	O_(Tyr166)_	L_eq_	L_ax_
Water	1.95	2.05	1.97	2.44	2.07	2.39
Glycine	1.96	2.06	1.97	2.35	2.01	2.80
Asparagine	1.96	2.03	2.01	2.41	1.95	3.01
Phenylalanine	1.96	2.04	1.95	2.34	2.00	3.90 *
Histamine	2.01	2.06	2.04	2.79	2.04	2.19
Pyruvic acid *^a^*	1.95	2.08	1.98	2.81	2.43	1.98
Glyoxylic acid *^b^*	1.97	2.05	1.99	2.51	1.96	2.53
Glyoxylic acid *^a^*	1.96	2.07	1.97	2.36	1.96	- **
Pyruvic acid *^b^*	1.97	2.04	1.99	2.63	1.96	2.41
Histidine *^c^*	2.01	2.06	2.04	2.83	2.00	2.20
Histidine *^d^*	1.94	2.05	1.96	2.71	1.91	- **
Oxalic acid	1.98	2.07	2.02	3.02	1.98	2.15
Citric acid	1.98	2.06	2.01	3.36	1.95	2.16

*^a^* Binding pose A; *^b^* binding pose B; *^c^* ligand linked through C-atom; *^d^* ligand linked through N-atoms; * average distance to phenyl ring carbons; ** position not occupied.

## Data Availability

The data are contained within the article and Supplementary Materials.

## Supplementary Materials

The following supporting information can be downloaded at: https://www.mdpi.com/article/10.3390/antiox11061096/s1, Figure S1: Oxygen consumption in the turbidimetric assay of LPMO; Figure S2: Thermodynamic scheme of an inhibitor binding to a catalytic site; Figure S3: NPA spin density in the active site of LPMO in the oxidized state; Figure S4: Structures of the studies inhibitory complexes; Figure S5: Dependence of the binding free energy on the interaction energy; Table S1: Specific activities for LPMO determined with the AmplexRed activity assay in the presence of inhibitor oxalate; Table S2: Cello-oligomers produced by NcLPMO9C in the presence of different inhibitors; Table S3: Atomic charges and spin densities calculated by NPA; Table S4: Total binding energies and free binding energies; Table S5: Major contributions to ligand binding according to the NBO analysis.

## Abbreviations

[2,6-DMP]	2:6-dimethoxyphenol
[hydrocoerulignone]	3,3′,5,5′-tetramethoxy-4,4′-dihydroxybiphenyl
[coerulignone]	3,3′,5,5′-tetramethoxydiphenoquinone
[BSSE]	Basis set superposition error
[DEE]	Diethyl ether
[DMSO]	Dimethylsulfoxide
[H_2_O_2_]	Hydrogen peroxide
[LPMO]	Lytic polysaccharide monooxygenase
[MF]	Molecular dynamics
[NBO]	Natural bond orbital
[NPA]	Natural population analysis
[PASC]	Phosphoric-acid-swollen cellulose
[QM]	Quantum mechanical
[NaOH]	Sodium hydroxide
[SD]	Standard deviation
[SE]	Standard error
