# Supplementary material for: Inhibition of the Peroxygenase Lytic Polysaccharide Monooxygenase by Carboxylic Acids and Amino Acids
Source: Antioxidants (Basel). 2022 May 31;11(6):1096. doi: 10.3390/antiox11061096 (PMC9220355; doi:10.3390/antiox11061096)
Supplement: Supplementary file 1 [file antioxidants-11-01096-s001.zip › antioxidants-1705169-supplementary.pdf]

SUPPLEMENTARY MATERIALS

Inhibition of the peroxygenase lytic polysaccharide monooxygenase by carboxylic acids and amino acids

Erik Breslmayr[1,2], Peter Poliak[2,3], Alen Pozgajcic[1,4], Roman Schindler[1], Daniel Kracher[1,5*], Chris Oostenbrink[2] & Roland Ludwig[1]

[1] Institute of Food Technology, Department of Food Science and Technology, University of Natural Resources and Life Sciences (BOKU), 1190 Vienna, Austria

[2] Institute of Molecular Modeling and Simulation, University of Natural Resources and Life Sciences (BOKU), 1190 Vienna, Austria

[3] Department of Chemical Physics, Faculty of Chemical and Food Technology, Slovak University of Technology, 812 37 Bratislava, Slovakia

[4] Department of Biochemical Engineering, Faculty of Food Technology and Biotechnology, University of Zagreb, 10000 Zagreb, Croatia

[5] Institute of Molecular Biotechnology, Graz University of Technology, Petersgasse 14, 8010 Graz, Austria

Erik Breslmayr: erik.breslmayr@boku.ac.at
Peter Poliak: peter.poliak@boku.ac.at
Alen Pozgajcic: alen.pozgajcic@gmail.com
Roman Schindler: roman.schindler@students.boku.ac.at
*Daniel Kracher: daniel.kracher@tugraz.at
Chris Oostenbrink: chris.oostenbrink@boku.ac.at
Roland Ludwig: roland.ludwig@boku.ac.at
*corresponding author

Contents

Supplementary figures S1–S5

Supplementary tables S1–S5

Supplementary figures

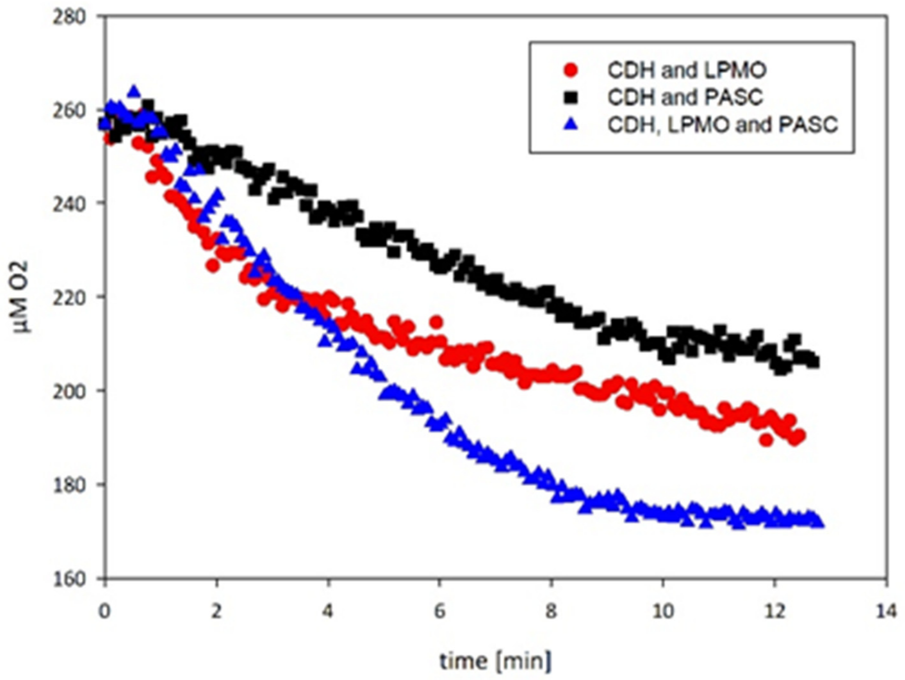

Figure S1. Oxygen consumption in the turbidimetric assay of LPMO. In the presence of only CDH and substrate (cellobiose and PASC, black squares) H_2O_2 is produced. CDH and LPMO, in the absence of PASC (red circles), increase H_2O_2 production by the LPMO uncoupling reaction. CDH and LPMO in the presence of PASC (blue triangles) show the same oxygen consumption rate (to CDH and LPMO in the absence of PASC) in the beginning but does not show the reduced activity after 3 min.

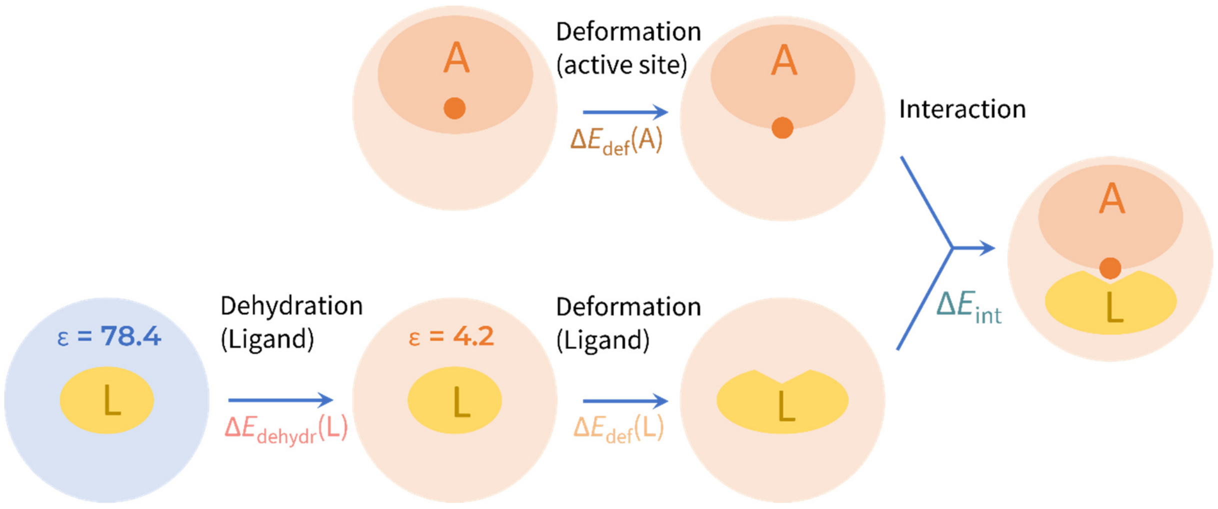

Figure S2. Thermodynamic scheme of an inhibitor binding to a catalytic site.

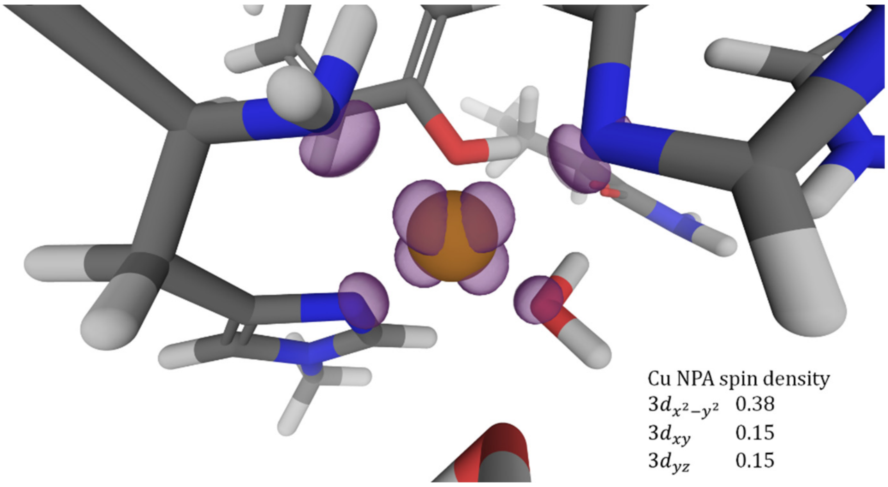

Figure S3. NPA spin density (isovalue = 0.01) in the active site of LPMO in the oxidized state.

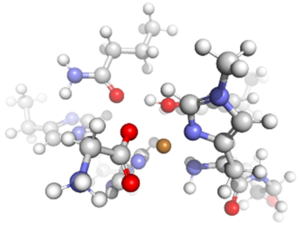

a) glycine

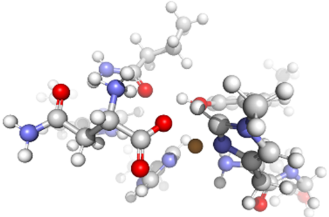

b) asparagine

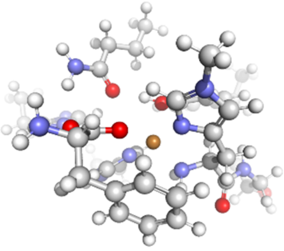

c) phenylalanine

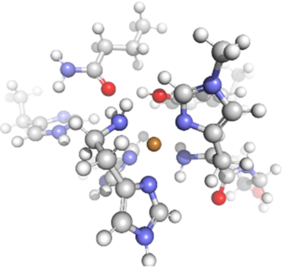

d) histamine

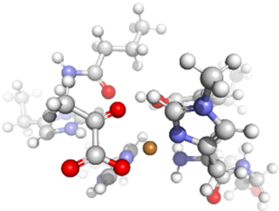

e) Pyruvic acid – pose A

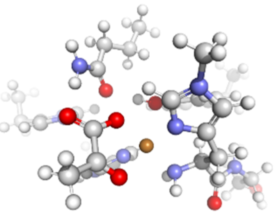

f) Pyruvic acid – pose B

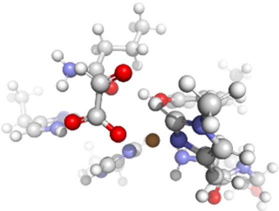

g) Glyoxylic acid – pose A

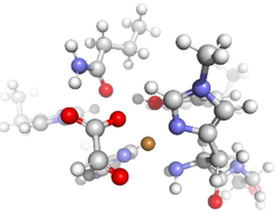

h) Glyoxylic acid – pose A

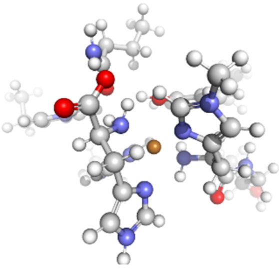

i) Histidine – N-linked

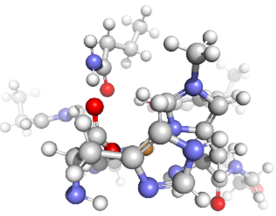

j) Histidine – C-linked

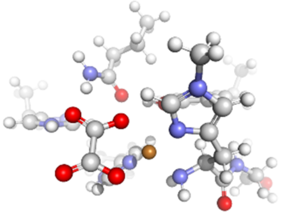

k) Oxalic acid

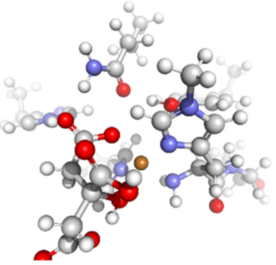

l) Citric acid

Figure S4. Structures of the studied inhibitory complexes.

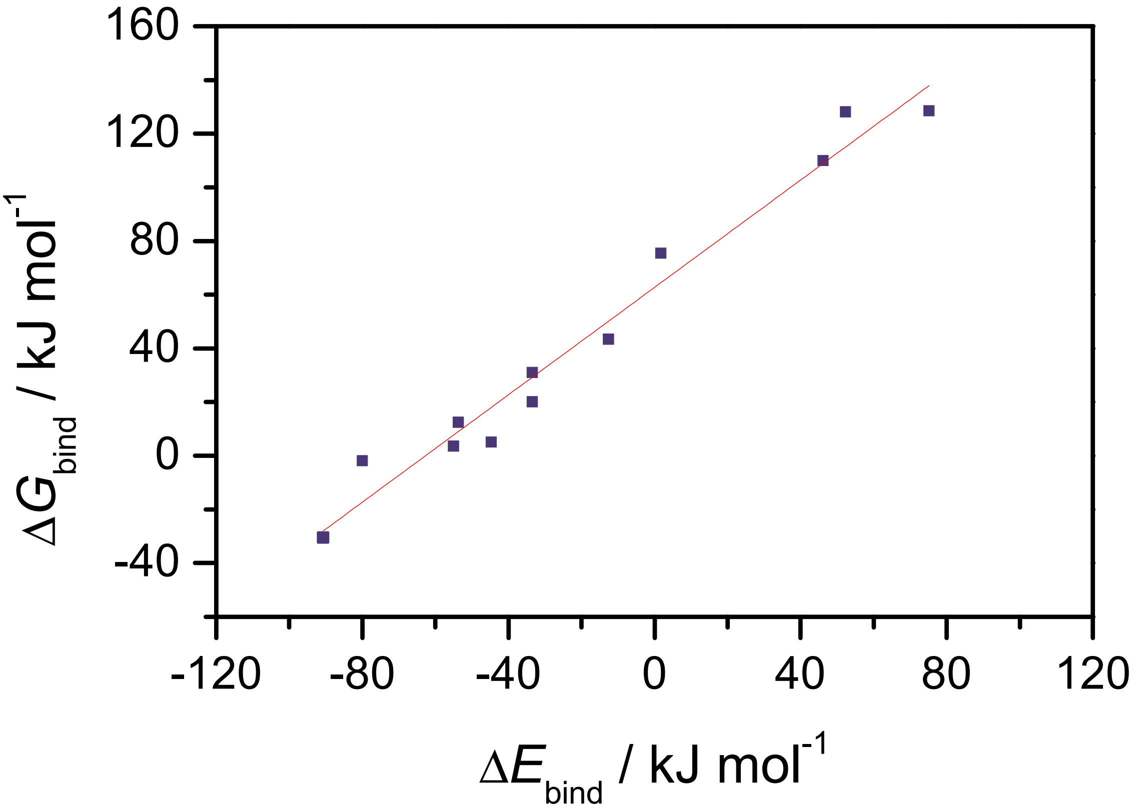

Figure S5. Dependence of the binding free energy on the interaction energy.

Supplementary tables

Table S1. Specific activities for LPMO determined with the AmplexRed activity assay in the presence of inhibitor oxalate. Conditions: 1 µM *Nc*LPMO9C, 1 µM *Nc*CDHIIA, 10 mM lactose, 50 µM AmplexRed, 7 U mL^{-1} horseradish peroxidase.

buffer	*Nc*LPMO9C - background	*Nc*LPMO9C	Background
	Specific activity (U g^{-1})		
acetate	4.9 ± 0.1	7.5 ± 0.1	2.6 ± 0.1
oxalate	0.1 ± 0.1	3.5 ± 0.1	3.4 ± 0.1

Table S2. Cello-oligomers produced by *Nc*LPMO9C in the presence of different inhibitors.

	**cellobiose**		**cellotriose**		**cellotetraose**		**cellopentaose**		**cellohexaose**		**Total**
	**µM**	**SD**	**µM**	**SD**	**µM**	**SD**	**µM**	**SD**	**µM**	**SD**	**µM**
					+ Ascorbic acid						
Acetic acid	16.1	0.9	18.3	1.8	8.9	0.1	-	-	-	-	**43.3**
Lactic acid	16.2	0.1	22.6	0.3	11.2	0.1	-	-	-	-	**50.0**
Pyruvic acid	33.7	3.1	23.1	1.7	10.7	0.2	-	-	-	-	**67.5**
Oxalic acid	44.1	2.3	29.6	0.5	11.3	0.1	-	-	0.5	0.1	**85.4**
Citric acid	59.3	0.6	38.4	0.1	12.7	0.1	-	-	0.5	0.1	**111.0**
Phosphoric acid	21.2	1.2	11.5	0.6	4.6	0.2	-	-	-	-	**37.3**
					- Ascorbic acid (blank)						
Acetic acid	4.2	0.3	4.3	0.3	6.2	0.1	-	-	-	-	**14.6**
Lactic acid	3.5	0.5	3.6	0.2	5.4	0.4	-	-	-	-	**12.4**
Pyruvic acid	5.6	0.5	3.3	0.2	-	-	-	-	-	-	**9.0**
Oxalic acid	6.8	0.4	3.7	0.3	5.3	0.5	-	-	-	-	**15.8**
Citric acid	7.2	0.8	4.2	0.1	5.9	0.2	-	-	-	-	**17.3**
Phosphoric acid	6.9	0.8	3.3	0.3	4.6	0.5	-	-	-	-	**14.8**

Table S3. Atomic charges and spin densities calculated by NPA [46] and equilibrial bond lengths of the *Nc*LPMO9c catalytic site.

			Cu-X bond length / Å	
	NPA atomic charge / a.u.	NPA spin density	DFT/B3LYP/6-31G(d)	X-ray
Cu	1.186	0.738		
$N_{imidazole(Me\text{-}His1)}$	-0.632	0.069	1.950	1.994
$N_{amine(Me\text{-}His1)}$	-0.925	0.089	2.045	2.113
$N_{(His83)}$	-0.621	0.062	1.972	2.029
$O_{(Tyr166)}$	-0.747	0.000	2.441	2.550
$O_{water(eq)}$	-0.968	0.043	2.071	2.183
$O_{water(ax)}$	-0.957	0.001	2.390	2.464

Table S4. Total binding energies and binding free energies in kJ mol^{-1}. Corresponding structures are shown in Figure S4.

	ΔE_{bind}	ΔG_{bind}
Water	61	174
Glycine	39	128
Asparagine	17	128
Phenylalanine	6	110
Histamine	-14	75
Pyruvic acid[a]	-76	43
Glyoxylic acid[b]	-94	20
Glyoxylic acid[a]	-94	31
Pyruvic acid[b]	-108	5
Histidine[c]	-131	12
Histidine[d]	-157	-2
Oxalic acid	-255	4
Citric acid	-267	-31

[a] binding pose A
[b] binding pose B
[c] ligand linked through C-atom
[d] ligand linked through N-atoms

Table S5. Major contributions to ligand binding according to the NBO analysis ($E^{(2)}$ above 17.0 kJ mol^{-1}).

				Principal NBO delocalizations		2nd order perturbative estimate	
	Principal bond	Bond length / Å	Wiberg bond order	Donor	Acceptor	$E^{(2)}$ (α)	$E^{(2)}$ (β)
$(H_2O)_2$	Cu - $O_{water,eq}$	2.071	0.196	197. n_O: $sp^{1.88}$	192. n_{Cu}*: s	57.4	67.4
				197. n_O: $sp^{1.88}$	194. n_{Cu}*: $sp^{1.00}$	39.6	37.2
	O_{Gln} – H_{water} – O_{water}	1.735	0.074	182. $n_{O(Gln)}$: $sp^{0.68}$	853. $\sigma_{O-H(water)}$*: $sp^{2.36}$	28.1	27.9
	Cu - $O_{water,ax}$	2.390	0.1368	199. n_O: $sp^{1.67}$	195. n_{Cu}*: p	38.3	39.9
				199. n_O: $sp^{1.67}$	192. n_{Cu}*: s	27.2	26.5
Glycine	Cu - O_{Gly}	2.007	0.238	207. n_O: $sp^{5.72}$	200. n_{Cu}*: s	65.1	72.0
				207. n_O: $sp^{5.72}$	199. n_{Cu}*: d(β)	0.0	37.4
				206. n_O: $sp^{1.14}$	201. n_{Cu}*: p	24.1	26.1
				206. n_O: $sp^{1.14}$	200. n_{Cu}*: s	18.5	22.8
Oxalate	Cu - $O_{oxal,eq}$	1.978	0.265	206. n_{O101}(α,2): $sp^{2.83}$	198. n_{Cu}*(α,6): s	75.4	62.3
				206. n_{O101}(α,2): $sp^{2.83}$	201. n_{Cu}*(α,9): p	59.8	0.0
				205. n_{O101}(α,1): $sp^{1.81}$	201. n_{Cu}*(α,9): p	36.4	0.0
				206. n_{O101}(β,2): $sp^{9.28}$	197. n_{Cu}*(β,5): d	0.0	41.4
				205. n_{O101}(β,1): $sp^{0.96}$	201. n_{Cu}*(β,9): p	0.0	34.4
				205. n_{O101}(β,1): $sp^{0.96}$	198. n_{Cu}*(β,6): s	0.0	26.4
				207. n_{O101}(β,3): p	198. n_{Cu}*(β,6): s	0.0	25.9
				207. n_{O101}(β,3): p	197. n_{Cu}*(β,5): d	0.0	25.8
				206. n_{O101}(β,2): $sp^{9.28}$	200. n_{Cu}*(β,8): p	0.0	23.8
				205. n_{O101}(β,1): $sp^{0.96}$	200. n_{Cu}*(β,8): p	0.0	22.0
	Cu - $O_{oxal,ax}$	2.146	0.231	203. n_{O100}(2): $sp^{3.47}$	198. n_{Cu}*(α,6): s	60.4	57.7
				203. n_{O100}(α,2): $sp^{3.47}$	200. n_{Cu}*(α,8): p	46.4	0.0
				202. n_{O100}(α,1): $sp^{1.50}$	200. n_{Cu}*(α,8): p	22.8	0.0
				203. n_{O100}(β,2): $sp^{3.46}$	201. n_{Cu}*(β,9): p	0.0	27.2
				203. n_{O100}(β,2): $sp^{3.46}$	200. n_{Cu}*(β,8): p	0.0	23.1
				202. n_{O100}(β,1): $sp^{1.50}$	201. n_{Cu}*(β,9): p	0.0	21.4
	O_{104} - H - (N_{Gln})	1.857	0.0696	209. n_{O104}(2): p	877. $\sigma_{N-H(Gln)}$*: $sp^{2.04}$	29.0	28.0
